# Using theories of behaviour to understand transfusion prescribing in three clinical contexts in two countries: Development work for an implementation trial

**DOI:** 10.1186/1748-5908-4-70

**Published:** 2009-10-24

**Authors:** Jill J Francis, Alan Tinmouth, Simon J Stanworth, Jeremy M Grimshaw, Marie Johnston, Chris Hyde, Charlotte Stockton, Jamie C Brehaut, Dean Fergusson, Martin P Eccles

**Affiliations:** 1 Health Services Research Unit, University of Aberdeen, UK; 2 Clinical Epidemiology Programme, Ottawa Hospital Research Institute and Department of Medicine, University of Ottawa, Canada; 3 Canadian Blood Services, Ottawa, Canada; 4 NHS Blood & Transplant, John Radcliffe Hospital, Headington, Oxford, UK; 5 Department of Psychology, University of Aberdeen, UK; 6 Centre for Medical Statistics and Health Evaluation, University of Liverpool, UK; 7 Clinical Epidemiology Programme, Ottawa Hospital Research Institute and Department of Epidemiology and Community Medicine, University of Ottawa, Canada; 8 Institute of Health and Society, Newcastle University, UK

## Abstract

**Background:**

Blood transfusion is an essential part of healthcare and can improve patient outcomes. However, like most therapies, it is also associated with significant clinical risks. In addition, there is some evidence of overuse. Understanding the potential barriers and enablers to reduced prescribing of blood products will facilitate the selection of intervention components likely to be effective, thereby reducing the number of costly trials evaluating different implementation strategies. Using a theoretical basis to understand behaviours targeted for change will contribute to a 'basic science' relating to determinants of professional behaviour and how these inform the selection of techniques for changing behaviour. However, it is not clear which theories of behaviour are relevant to clinicians' transfusing behaviour. The aim of this study is to use a theoretical domains framework to identify relevant theories, and to use these theories to identify factors that predict the decision to transfuse.

**Methods:**

The study involves two steps: interview study and questionnaire study. Using a previously identified framework, we will conduct semi-structured interviews with clinicians to elicit their views about which factors are associated with waiting and further monitoring the patient rather than transfusing red blood cells. Interviews will cover the following theoretical domains: knowledge; skills; social/professional role and identity; beliefs about capabilities; beliefs about consequences; motivation and goals; memory, attention, and decision processes; environmental context and resources; social influences; emotion; behavioural regulation; nature of the behaviour. The interviews will take place independently in Canada and the UK and involve two groups of physicians in each country (UK: adult and neonatal intensive care physicians; Canada: intensive care physicians and orthopaedic surgeons). We will: analyse interview transcript content to select relevant theoretical domains; use consensus processes to map these domains on to theories of behaviour; develop questionnaires based on these theories; and mail them to each group of physicians in the two countries. From our previous work, it is likely that the theories will include: theory of planned behaviour, social cognitive theory and the evidence-based strategy, implementation intention. The questionnaire data will measure predictor variables (theoretical constructs) and outcome variables (intention and clinical decision), and will be analysed using multiple regression analysis. We aim to achieve 150 respondents in each of the four groups for each postal survey.

## Background

### Blood transfusion

Blood transfusion is an essential part of modern healthcare, and can improve patient outcomes. However, like most therapies, it is also associated with significant clinical risks [1]. It is also a scarce and costly resource [2]. There is published evidence of variation and suboptimal clinical transfusion practice in all aspects of transfusion. For example, there is ongoing evidence of large differences between hospitals and clinical teams in the use of red cell transfusions (and other blood products) for the same surgical procedures with no clear explanation based on clinical factors, *e.g.*, pre-operative haemoglobin, peri-operative blood loss [3]. In a large multicentre study of Canadian intensive care unit (ICU) patients transfused over a two-year period, the percentage of patients transfused varied from 20% to 54% across 11 different centres [4]. A recent regional audit in the UK in patients undergoing primary hip replacement surgery showed a range of 23% to 58% in the proportion of patients who were transfused [2]. The majority of patients who were transfused received only one or two units of blood, and most were discharged with a haemoglobin concentration above 10 g/dl. If they had not been transfused, they would still have had a haemoglobin concentration above 8 g/dl, which would not be expected to impair postoperative recovery [5]. Some teams even carry out major procedures without blood transfusion by attention to patient care throughout the peri-operative period [6]. The combination of algorithms for blood management and restrictive transfusion thresholds may in these situations offer a more effective approach to blood conservation than the implementation of single interventions such as autologous transfusion [7].

### Changing transfusion practice

Variation in the use of blood suggests the potential for a reduction in blood usage without having a negative impact on patient care. Many different forms of behaviour change interventions are used in current clinical practice by hospitals and blood transfusion services worldwide with the aim of changing transfusion practice, including the dissemination of guidelines, retrospective or prospective audits, educational events, and algorithms for blood prescribing [8,9]. A systematic review found that all interventions for the reduction of transfusion studied in clinical trials seemed to be effective, with a reduction in inappropriate transfusions of 12% to 83% [8] and of 9% to 77% for the total number of units transfused [9]. However, there were significant limitations to the quality of this evidence. Most of the studies were uncontrolled before-and-after studies that are prone to secular changes, maturation bias, and bias in favour of the intervention. Most were single-centre studies, and many were performed more than 10 years ago. The universal success in the published studies raises the possibility of publication bias. These concerns about the true effectiveness and the durability of the effect of these interventions were raised by one study, which reported a return to the baseline rate of transfusions three months after the completion of the intervention, and a follow-up report from another study [10,11] that reported a return to previous transfusion practice. Finally, none of the studies formally reported cost-effectiveness comparisons.

### Implementation research

Although the transfer of research findings into practice is often slow, quality improvement research [12-14] has demonstrated that interventions can be effective, although providing less information to guide the choice or to optimise the components of complex interventions in actual practice [15]. Hence, there is still a need to study the influences on healthcare professionals' behaviour and to design interventions to enable them to use research findings more effectively. A range of factors (*e.g.*, patient preference, paucity of solid evidence on which clinicians can base their decisions, availability of institutional or jurisdictional protocols or guidelines) may influence transfusion practice and interventions to change clinical practice can be aimed at a number of levels (individual health care professionals, health care groups or teams, organisations providing health care, the larger health care system, or environment). While the majority of interventions have been aimed at individual practitioners, the study described in this protocol will be based on a broad theoretical framework that includes potential predictors from models of individual, team-level, and organisational behaviour. Nevertheless, the outcome to be predicted will be the behavioural intention and simulated behaviour (specific clinical decisions) of individual clinicians, as ultimately it is the individual clinician who decides much of the face-to-face health care.

### The use of behavioural theory in changing the behaviour of healthcare professionals

While the effectiveness of interventions varies across different clinical problems, contexts, and organisations, studies in general have provided scant theoretical or conceptual rationale for their choice of intervention [16] and only limited descriptions of the interventions and contextual data.

The UK Medical Research Council (MRC) has proposed a framework for developing and evaluating complex interventions [17,18]. The revised framework [18] gives prominence to the development of the intervention, which includes identifying the evidence base, identifying or developing theory, and modelling the process or outcomes. We interpret these three research activities for implementation research as follows:

1. Identifying the evidence base encompasses two distinct components in implementation research: verification of an evidence-practice gap (*i.e.*, would quality of health care be improved if clinicians' behaviour changed?), and identification of specific evidence-based behaviour change techniques ('active ingredients') that are likely to lead to change.

2. Identifying or developing theory is essential for understanding the likely mechanisms of behaviour change. This informs questions such as how best to deliver and optimise the techniques.

3. Modelling the process or outcomes involves an empirical investigation by which the theorised pathways that change or maintain the current behaviour are tested in a real or simulated clinical context. Results of this investigation feed back into the evidence base relating to the behaviour change techniques as well as confirming, disconfirming, or developing theory.

The MRC framework thus proposes a systematic, theory- and evidence-based approach to the identification of the 'active ingredients' for achieving professional behaviour change. By using a theoretical basis to identify determinants of behaviour and to select behaviour change interventions, the chances of finding a significant improvement in response to a specific set of interventions should increase, thereby reducing the number of costly trials evaluating different implementation strategies. Additionally, this approach should allow for the combination of complementary techniques, and possibly identification of clinical contexts that require different approaches to target different groups of physicians.

However, this first requires the selection of an appropriate theoretical framework. A number of psychological theories have been used to explore the factors associated with the behaviours of health care practitioners, *e.g.*, the theory of planned behaviour (TPB) [19] and social cognitive theory [20]. Psychological theories are numerous, and, in order to rationalise their use, a consensus group of UK health psychologists derived 12 theoretical 'domains' that incorporated all relevant theories, and that could be used to investigate the implementation of evidence-based practice [21]. A Table reproducing these domains and the theoretical constructs they include is shown in Additional File 1. These theoretical domains offer a comprehensive list of potentially relevant constructs for the behaviour of transfusing. They include factors such as local protocols, management policies, and resources available in the local context. Based on these constructs, it may be possible to adopt a systematic approach to selecting appropriate theories.

### Clinical Contexts

The target patient groups for this investigation are patients in ICUs, patients in neonatal intensive care units (NICUs), and orthopaedic patients (*e.g.*, undergoing surgery for hip fracture). While medical specialties represent significant users of red blood cells, as a broad generalisation, transfusion practices in medicine and paediatrics have been less evaluated by comparison to surgical specialities in audits of transfusion. Within the medical specialties, neonates represent a clinical group in which patients can be heavily exposed to blood and blood components (*i.e.*, over 90% of extremely low birth weight infants are treated with blood products) [22], and the risks of blood (including variant Creutzfeldt-Jakob disease ) assume greater significance in view of potential for longer survival. The evidence base to guide transfusion practice is strongest in adult critical care, though still incomplete. The evidence base in neonates is slowly developing, and a number of recent randomised controlled trials have been published that, although reporting different findings, begin to inform and direct behaviour [22,23]. As in adult intensive care, there is the emergence of over-transfusion as a problem. In the UK, the PlaNeT study [24], a prospective cohort of over 150 enrolled severely thrombocytopenic neonates and 300 platelet transfusions administered to nearly 100 neonates at seven different NICUs in Southern and Eastern England, demonstrated that most transfused neonates are preterm, but they only require short term platelet transfusions for prophylaxis. This study has helped raise the awareness of transfusion and created a network of interested neonatologists. Orthopaedic surgery remains one of the largest users of red blood cells, as hip and knee replacement surgeries are relatively common procedures which have a high risk of anaemia. Yet there is little evidence to guide transfusion practice in orthopaedic surgery. However, at least one large randomized controlled trial in hip fracture patients is underway in the US with expected completion in 2009 [5].

### UK and Canadian blood services

The NHS blood and transplant (NHSBT) service is responsible for managing all the products and services, including promoting blood, tissue, and organ donation to the public; managing the supply of blood to hospitals in England and Wales; and working with hospital staff to promote the safe and appropriate use of blood. The Canadian Blood Services (CBS) performs many similar functions for nine provinces and three territories in Canada. Both the NHSBT and the CBS work at the hospital, regional, and national levels to promote appropriate use of blood and blood products. As such, they are critical partners in the UK and Canada for the development and testing of interventions to optimize transfusion practice.

The clinical evidence base to support more restrictive transfusion practice is fairly new and, to our knowledge, there have been no investigations of the factors that might hinder or facilitate change in clinicians' transfusion behaviour in these clinical contexts. To provide an opportunity to make cross-country comparisons while investigating a broad range of clinical contexts, we decided to design parallel studies in two clinical contexts in each country (Canada and the UK) and to select one clinical context (adult intensive care) for investigation in both countries.

### Aims and Objectives

The aim of the study is to use constructs from psychological theories to identify factors that predict the decision to transfuse. The objectives are:

1. To conduct semi-structured interviews (based on the theoretical domains framework) with transfusion prescribers in two clinical contexts in both the UK and Canada, to identify theoretical constructs (and therefore psychological theories) relevant to the use of red blood cells.

2. To develop a questionnaire based on the identified theories.

3. To conduct separate postal questionnaire surveys of intensive/critical care physicians and neonatal physicians in the UK, and intensive/critical care physicians and orthopaedic surgeons in Canada.

4. To identify the psychological constructs that, within a theoretical framework, predict the decision by neonatal and critical care consultants and orthopaedic surgeons to transfuse red blood cells (as opposed to continuing to monitor the patient).

## Methods

### Clinical setting

We will work with two groups of clinicians in each country -- intensive/critical care physicians and neonatal physicians in the UK and intensive/critical care physicians and orthopaedic surgeons in Canada. Physicians in intensive care are significant users of red blood cells, and there has been a landmark trial in which mortality was compared in two groups of patients randomised to receive red cells at a more or less restrictive haemoglobin trigger threshold [25,26]. In the UK, there is an evolving network of critical care physicians across Scotland, Wales, Northern Ireland, and England who have supported the ATTICS study (which demonstrated a narrowing but persistent gap in clinical practice and the evidence for red cell transfusions in critical care patients) [27-29] in Scotland and the intensive care study of coagulopathy (ISOC) (which described the current practice of frozen plasma transfusions in critical care patients [30]) in all four countries. It is expected that the heightened awareness of transfusion generated through these studies will facilitate a high response rate to the questionnaires planned in this survey. Similarly in Canada, there is a well established critical care research network (Canadian critical care trials group), which has carried out much of the seminal work on blood transfusions including the TRICC [26] and TRIPICU [32] trials evaluating restrictive transfusion triggers in adult and paediatric patients, respectively.

### Interview study

We will conduct interviews with clinicians from each clinical area, including physicians at regional centres/academic/teaching and district/community hospitals. Although convenience sampling will be used, participants will also be selected in such a way as to ensure diversity of age, gender, and hospital size so as to maximise the range of views expressed. Although the sample will thus not necessarily be representative, we will nevertheless identify a wide range of views for consideration in the design of the questionnaire.

The linguistic problem of double negatives makes it quite difficult to conduct interviews about 'not transfusing.' Preliminary discussions with consultants in the chosen contexts established that the alternative to transfusing is continued monitoring of the patient, or 'watching and waiting.' Using the previously identified framework [21], we will therefore ask a series of questions related to the practice of 'watching and waiting' rather than transfusing red blood cells. The interview will cover the following domains: knowledge; skills; social/professional role and identity; beliefs about capabilities; beliefs about consequences; motivation and goals; memory, attention, and decision processes; environmental context and resources; social influences; emotion; behavioural regulation; nature of the behaviour. We will not necessarily use these technical terms but will phrase the issues appropriately for the clinical context (see Additional File 2).

These interview sessions will be conducted by a trained interviewer. The sessions will be tape recorded and transcribed for analysis. We anticipate interviewing up to 10 clinicians from each group. The total number of interviews will be determined by the time it takes to reach data saturation such that no further specific beliefs will be gathered by additional interviews.

The interview transcripts will be analysed in two stages. In stage one, we will identify all domains in which clinicians report a wide range of beliefs or in which clinicians report problems when making the decision to watch and wait rather than transfuse red cells (This paper is now published [33]). A domain will be considered 'relevant' if frequently mentioned responses indicate that it might affect the decision to transfuse or not. The specific beliefs within each domain will also be coded. In stage two, the specific beliefs in each of the relevant domains will be used to identify constructs in the following manner. Independent judges will use the list of beliefs (from the stage one analysis) and a list of constructs included in each theoretical domain (Additional File 1) to match the beliefs with the constructs. The identified constructs from the relevant domains will be discussed by the research team to agree the theoretical models that represent these constructs. If the identified constructs represent part of a theory, and are not represented in other identified theories, then the entire theory will be included for operationalising in the questionnaire study.

### Questionnaire study

#### Study design

The predictor variables will be the constructs within the psychological theories selected in the interview study. The outcome variables will be behavioural intention and simulated behaviour for 'watching and waiting instead of transfusing red blood cells.'

#### Questionnaire content

Based on the interview study results, we will use the theories that most clearly represent the construct domains identified and that can be operationalised in a questionnaire format. From our previous work, it is likely that the following theories (that have been evaluated in other health care settings) will be included: the TPB [19], social cognitive theory [20] and the evidence-based strategy, implementation intention [34]. These theories have been examined thoroughly in other health care settings, they include explanatory factors that are amenable to change, and they include non-volitional components which assume that individuals do not always have complete control over their actions (*e.g.*, the impact of insufficient financial resources). Table 1 contains examples of the variables within the theories and example questions, and Additional File 3 shows an example of a finished instrument used for the UK study of intensive care consultants.

**Table 1:** Examples of likely relevant theories, the variables within the theories, and questionnaire items for the behaviour of managing patients with borderline haemoglobin by watching and waiting instead of transfusing red cells.

**Theory**	**Predictor Variables**	**Illustrative items**
Theory of Planned Behaviour [22]	Attitude Subjective norm (perceived pressure) Perceived behavioural control; Intention	In general, the benefits of managing patients with borderline haemoglobin by watching and waiting instead of transfusing red cells outweigh the harms. (Attitude)

Social Cognitive Theory [23]	Self-efficacy Goals relevant to watching and waiting	I am confident that I can manage a patient with borderline haemoglobin by watching and waiting instead of transfusing red cells. (Self-efficacy)

Implementation Intention [36]	Action plan	I have a clear plan of how I will manage patients with borderline haemoglobin by watching and waiting instead of transfusing red cells.

#### Measures

To evaluate behavioural intention (to watch and wait instead of transfusing red blood cells) and its possible predictors, the questionnaire will include questions with multi-item scales to assess elements of the identified psychological theories. Responses will be elicited using a seven-point Likert scale. The content for these questions assessing specific beliefs regarding the practice of watching and waiting rather than transfusing with red blood cells will be derived from standard practice (*e.g.*, [19,35]) and from the interviews.

We will use three proxy measures of behaviour -- strength of intention, 'direct estimation' of intention, and simulated behaviour (see Additional File 3).

The questionnaire will measure simulated behaviour. A number of clinical scenarios will be developed for each clinical group. The clinical scenarios will use different combinations of patient characteristics (*e.g.*, diagnosis, co-morbidities, and laboratory results), and for each scenario, physicians will be asked to report their clinical decision about whether to transfuse. Examples of scenarios are attached (Additional File 3).

#### Sampling procedure

With permission from the relevant professional societies, we will select a random sample of names from lists of practising consultants in each country.

#### Implementation of the postal questionnaires

A covering letter, which will explain the purpose and nature of the study, will be included with the initial copy of the questionnaire. The letter will explain the importance of completing the questionnaire, outline how the results will be used, and ensure the confidentiality of the responses. A reminder letter will be sent at week two with an additional copy of the questionnaire.

#### Analysis and sample size

The questionnaire data will allow us to assess the relationships between predictor (theoretical constructs) and three outcome measures (strength of intention, direct estimation of intention, and simulated behaviour). These relationships will be assessed using multiple regression analysis. Sample size calculations for the multiple regression analysis depend on the number of cases per predictor variable [36]. A minimum sample size of 50 + 8 m is required, where m is the number of predictor variables required for the multiple regression analysis. To test individual predictors, a sample size of 104 + m is required. We estimate approximately 12 predictor variables, which will require 146 respondents to test for multiple correlations, and 116 to test individual predictor variables. We will aim to achieve 150 respondents for each of the two surveys in each country (*i.e.*, 600 respondents in all). Assuming a response rate of 50%, we will send the questionnaire to 300 consultants in each group. To maximise the response rate, a customised letter inviting consultants to participate will be signed by a clinical member of the research team who is well known within each clinical specialty. As the consultant groups in each of the countries are close-knit professional bodies, we expect a high level of support for this work and thus reasonably high response rates.

#### Project advisory group

We will establish a project advisory group in each country that will contain clinical as well as academic representatives and international collaborators. It will meet at intervals dictated by the work.

#### International comparisons

The studies in the two countries will be run independently through the steps outlined previously. By using methods in common, we will have the opportunity to compare results for physicians working in similar clinical settings in two different countries, and working in two different clinical settings within the same country.

We will use multivariate analysis of variance to compare mean scores on each construct (*e.g.*, to identify any differences between national or clinical context in intention), and multiple regression approaches to identify whether national or clinical context adds to the prediction of intention and simulated behaviour. Specifically, we will investigate the following comparisons: intensive care consultants (UK and Canada); intensive care consultants and orthopaedic surgeons (within Canada); intensive care consultants and neonatologists (within UK). This will add an important dimension to the interpretation of the results of this project as it will enable us to identify whether relevant domains, theories, and patterns of prediction converge or diverge across different health care systems and clinical contexts.

## Discussion

This study will be based on a theoretical domains framework that is derived from theories of behaviour to identify relevant theories, and use the theories to predict intention and simulated behaviour.

Conducting semi-structured interviews (based on the theoretical domains framework) to identify theoretical constructs (and therefore psychological theories) is an approach that has rarely been used before, and to our knowledge has not been used with respect to transfusion practice. The broad objective of this approach is to develop an evidence base for selecting theories that are relevant to a particular clinical behaviour in a specific context and that are thus likely to inform the design of studies seeking to predict or change those behaviours. Although this is likely to minimise the use of researchers' 'favourite' theories (whether or not they are relevant), there are potential pitfalls of this approach. First, if many theories are potentially relevant, then studies based on them are likely to become unwieldy and to lack the parsimony that is scientifically and pragmatically desirable. Second, the efficiencies gained by using the most relevant theories may be lost in the time and resources taken to conduct the kind of interview study proposed here. Nevertheless, we feel it is worth considering whether a systematic method for selecting theories is preferable to the use of favourite theories, or hunches about what might be relevant.

The questionnaire study will identify the psychological constructs that predict the decision by clinicians to transfuse red blood cells (as opposed to continuing to monitor the patient). Identifying the predictors of generalised intention about transfusing (or not) and simulated behaviour (*i.e.*, the decision whether or not to transfuse given a specific clinical scenario) will inform the development of interventions to increase evidence-based behaviour in these clinical contexts. It is therefore important to identify whether the prediction of intention is an appropriate basis for interventions to change actual behaviour.

Intention has been defined as 'indications of how hard people are willing to try, or how much effort they are planning to exert, in order to perform a behavior' [19]. A pre-requisite of this approach is that the interim endpoint (*e.g.*, measure of intention) must be predictive of real world outcomes. This is the case for behavioural intention in non-clinical populations as demonstrated by reviews of both observational and experimental studies. Godin and Kok [37] reported averaged correlations between intention and different health-related behaviours ranging from 0.35 to 0.56 (*i.e.*, intention was accounting for between 12% and 31% of the variance in behaviour). Armitage and Connor [38], from 63 independent studies reporting prospectively measured behavioural data, reported that the TPB variables that directly influence behaviour (intention and perceived behavioural control) accounted for a similar proportion of the variance in behaviour. When behaviour measures were self-reported, the TPB accounted for more of the variance in behaviour than when behaviour measures were objective or observed. A meta-analysis of 10 meta-analyses by Sheeran [39] reported that intention accounted for almost one-third of the variance in behaviour. Finally, Webb and Sheeran [40] reviewed experimental studies to relate change in intention to change in behaviour. From meta-analysis of 47 experimental tests of the intention-behaviour relationship, they concluded that a 'medium-to-large' change in intention leads to a 'small-to-medium' change in behaviour. Eccles *et al. *[41] and Godin *et al. *[42] have demonstrated that a similar relationship is apparent in the smaller number of available studies of healthcare professional behaviour. These reviews demonstrate that there is a reliable, but not perfect, relationship between stated intention and behaviour.

Considerable research effort has been directed to addressing the 'intention-behaviour gap,' and two approaches have been proposed. One addresses the variability of the link by focusing on moderators of the intention-behaviour relationship, such as intention certainty and attitudinal versus normative control [43,44]. According to this approach, it is possible to predict which individuals will enact their intentions (*e.g.*, those whose intentions are attitudinally controlled). A second approach focuses on mediators of the intention-behaviour relationship, or processes that might be regarded as 'post-intentional,' such as implementation intention [34]. This approach identifies processes that assist individuals to enact their intentions, thereby minimising the size of the intention-behaviour gap. Our approach will allow us to explore both approaches.

## Future work

This study corresponds to specific aspects of the UK's revised MRC framework for developing complex interventions [18], namely, identifying relevant theory and modelling process and outcome. The results of this project will be used to develop one or more interventions to change clinicians' behaviour. These will be piloted and then tested in a multi-site cluster randomised controlled trial.

## Competing interests

The authors declare that they have no competing interests.

## Authors' contributions

AT, SJS, MPE, and JMG conceived the study and, with CH, acquired funding. JJF, MJ, JMG, and MPE were theoretical and methodological advisers. All authors advised on clinical and methodological issues, provided ongoing critique and approved the final version of the manuscript.

## Supplementary Material

Additional file 1 **Theoretical domains and their component constructs from psychological theories used to understand transfusion prescribing (from ‘Psychological’ Theory Group)
** Click here for file

Additional file 2 **Theoretically-informed topic guide (core questions to elicit views within each theoretical domain, with follow-up prompts for guidance if needed) as used in interviews with critical care consultants for the UK study
** Click here for file

Additional file 3 **Postal questionnaire used in UK study of intensive/critical care consultants**.
Click here for file
