# Supplementary material for: Using theories of behaviour to understand transfusion prescribing in three clinical contexts in two countries: Development work for an implementation trial
Source: Implement Sci. 2009 Oct 24;4:70. doi: 10.1186/1748-5908-4-70 (PMC2777847; doi:10.1186/1748-5908-4-70)
Supplement: Additional file 1 — Theoretical domains and their component constructs from psychological theories used to understand transfusion prescribing (from ‘Psychological’ Theory Group) [file 1748-5908-4-70-S1.doc]

**Additional file 1:**  Theoretical domains and their component constructs [24].

| **Domains** | **Constructs** |
| --- | --- |
| (1) Knowledge | Knowledge  Knowledge about condition/scientific rationale  Schemas, mindsets and illness representations  Procedural knowledge |
| (2) Skills | Skills  Competence/ability/skill assessment  Interpersonal skills  Coping strategies |
| (3) Social/  professional role and identity | Identity  Professional identity/boundaries/role  Group/social identity  Social/group norms  Alienation/organisational commitment |
| (4) Beliefs about capabilities | Self-efficacy  Control – of behaviour and material and social environment  Perceived competence  Self-confidence/professional confidence  Empowerment  Self-esteem  Perceived behavioural control  Optimism/pessimism |
| (5) Beliefs about consequences | Outcome expectancies  Anticipated regret  Appraisal/evaluation/review  Consequences  Attitudes  Contingencies  Reinforcement/punishment/consequences  Incentives/rewards  Beliefs  Unrealistic optimism  Salient events/sensitisation/critical incidents  Characteristics of outcome expectancies – physical, social, emotional; sanctions/rewards, proximal/distal, valued/not valued, probable/improbable, salient/not salient, perceived risk/threat |
| (6) Motivation and goals | Intention; stability of intention/certainty of intention  Goals (autonomous, controlled)  Goal/target setting  Goal priority  Intrinsic motivation  Commitment  Distal and proximal goals  Transtheoretical model and stages of change |
| (7) Memory, attention and decision processes | Memory  Attention  Attention control  Decision making |
| (8) Environmental context and resources | Resources/material resources (availability and management)  Environmental stressors  Person x environment interaction  Knowledge of task environment |
| (9) Social influences | Social support  Social/group norms  Organisational development  Leadership  Team working  Group conformity  Organisational climate/culture  Social pressure  Power/hierarchy  Professional boundaries/roles  Management commitment  Supervision  Inter-group conflict  Champions  Social comparisons  Identity; group/social identity  Organisational commitment/alienation  Feedback  Conflict – competing demands, conflicting roles  Change management  Crew resource management  Negotiation  Social support: personal/professional/organisational, intra/interpersonal, society/community  Social/group norms: subjective, descriptive, injunctive norms  Learning and modelling |
| (10) Emotion | Affect  Stress  Anticipated regret  Fear  Burn-out  Cognitive overload/tiredness  Threat  Positive/negative affect  Anxiety/depression |
| (11) Behavioural regulation | Goal/target setting  Implementation intention  Action planning  Action planning  Self-monitoring  Goal priority  Generating alternatives  Feedback  Moderators of intention-behaviour gap  Project management  Barriers and facilitators |
| (12) Nature of the behaviour | Routine/automatic/habit  Breaking habit  Direct experience/past behaviour  Representation of tasks  Stages of change model |
