# Supplementary material for: Using theories of behaviour to understand transfusion prescribing in three clinical contexts in two countries: Development work for an implementation trial
Source: Implement Sci. 2009 Oct 24;4:70. doi: 10.1186/1748-5908-4-70 (PMC2777847; doi:10.1186/1748-5908-4-70)
Supplement: Additional file 2 — Theoretically-informed topic guide (core questions to elicit views within each theoretical domain, with follow-up prompts for guidance if needed) as used in interviews with critical care consultants for the UK study [file 1748-5908-4-70-S2.doc]

Theoretically-informed topic guide (core questions to elicit views within each theoretical domain, with follow-up prompts for guidance if needed) as used in interviews with critical care consultants for the UK study.

**INTOPT Interview Schedule**

**Introduction**

“The general aim of the study is to help us understand more about transfusion practice in Intensive Care Units. I want to do this by trying to figure out how you think when you’re making the decision about transfusing patients, by getting inside your head as it were. I’d like you to think about patients in general terms, having said that, I know it might be appropriate to think about specific patients or situations in order to answer certain questions. Some of these questions might be quite new to you, so I’d like you to give them some thought and answer frankly.”

How does that sound? Do you have any immediate comments/thoughts before we get started on the interview? Are you ready to get started?

“Certain studies have driven our research, one of which is the TRICC trial run by a Canadian group, Paul Hébert and colleagues, about different transfusion triggers. This trial had its good points, and like any other trial it had some difficulties.”

Are you aware of this study? What were your thoughts about it?

Did it influence your clinical practice?

Would you like me to leave you a copy of the paper? [if know nothing about the trial]

“As I said before, the evidence we have just talked about has driven the current study. As a result, I would like to go through the rest of the interview talking about your clinical practice in light of this evidence.

I am interested to hear your thoughts about how you might:
**manage a patient with borderline Hb** **by watching and waiting instead of transfusing RBCs.**

When I say borderline Hb, I’m thinking about those patients when the decision about transfusing might be difficult (not those you’d definitely transfuse or those you’d definitely not transfuse), you know, where there’s a bit of a grey area…**what would you consider borderline Hb?**

**KNOWLEDGE**

“We have talked about some of the evidence. I’d also like to find out about your knowledge and use of guidelines:”

Do you use any guidelines (to inform your transfusion practice)? If so, which ones? If not, for what reasons do you not use guidelines?

How do you use the guidelines, i.e. what do you actually, physically do? (behaviour – do you ever read the guidelines to check if a behaviour you performed was guideline compliant?)

How important do you think the guidelines are? ..and who do you think they are important for?

Are you happy with the way the guidelines you use reflect the evidence? (i.e. do the guidelines need updating?)

What other evidence are you aware of, or do you use?

(Do you think guidelines are a waste of time?)

**SOCIAL/PROFESSIONAL ROLE and IDENTITY**

Do you sometimes feel constrained by guidelines?...what about protocols?

How does this affect your professional autonomy?

Is there anything else about your professional role that influences how **you manage patients with borderline Hb by watching and waiting instead of transfusing RBCs**? (i.e. consensus in your profession)

Set the scene re: asking about “**managing a patient with borderline Hb by watching and waiting instead of transfusing RBCs**” – I’d like you to think about that for a moment…

**SOCIAL INFLUENCES**

Would other team members have a view of you **managing a patient with borderline Hb** **by watching and waiting instead of transfusing RBCs**?

What do you think those views might be?

How might the views of other team members affect you **managing a patient with borderline Hb** **by watching and waiting instead of transfusing RBCs**?

**BEHAVIOURAL REGULATION**

If you’re thinking about changing your own transfusion practice, how would you do this?

Are there procedures or ways of working that might encourage you to **manage a patient with borderline Hb** **by watching and waiting instead of transfusing RBCs**?

If you decided to **manage a patient with borderline Hb** **by watching and waiting instead of transfusing RBCs,** how confident are you that your team can carry this out?

**NATURE OF THE BEHAVIOURS**

“The evidence from research suggests that transfusion practice is variable. However, there is evidence to support a restrictive transfusion practice. With that in mind, in terms of **aiming to transfuse less**:”

What might need to be done differently?

What would you do differently?

Who needs to do what differently when, where, how, how often and with whom?

**SKILLS**

How easy or difficult would it be to **manage a patient with borderline Hb** **by watching and waiting instead of transfusing RBCs**?

What skills are required to **manage a patient with borderline Hb** **by watching and waiting instead of transfusing RBCs**?

**BELIEFS ABOUT CAPABILITIES**

How confident are you about doing this? (that you can **manage a patient with borderline Hb** **by watching and waiting instead of transfusing RBCs** (despite any difficulties)

What problems/difficulties do you think you might encounter in **managing a patient with borderline Hb** **by watching and waiting instead of transfusing RBCs**?

What would help you overcome these problems/difficulties?

**ENVIRONMENTAL CONTEXT and RESOURCES**

In what way is **managing a patient with borderline Hb** **by watching and waiting instead of transfusing RBCs** affected by different clinical situations? (any other situations?)

Are there any competing tasks or time constraints that influence whether you might **manage a patient with borderline Hb** **by watching and waiting instead of transfusing RBCs**?

**BELIEFS ABOUT CONSEQUENCES**

What are the benefits of **managing a patient with borderline Hb** **by watching and waiting instead of transfusing RBCs**? (to self, patients, colleagues, organisation – positive and negative, long-term and short-term, **financial**)

What are the disadvantages of **managing a patient with borderline Hb** **by watching and waiting instead of transfusing RBCs**? (to self, patients, colleagues, organisation – positive and negative, long-term and short-term, **financial**)

Are there any incentives to encourage **managing a patient with borderline Hb** **by watching and waiting instead of transfusing RBCs**?

**MOTIVATION AND GOALS**

How important do you feel it is to **manage a patient with borderline Hb** **by watching and waiting instead of transfusing RBCs**? (in relation to other behaviours required to treat the patient)

Would the goal of **managing a patient with borderline Hb** **by watching and waiting instead of transfusing RBCs** be incompatible with achieving some other objective?

**MEMORY, ATTENTION AND DECISION PROCESSES**

What thought processes might guide your decision to **manage a patient with borderline Hb** **by watching and waiting instead of transfusing RBCs**?

In what situations, if any, might it be difficult to think of alternatives to transfusing?

Is **managing a patient with borderline Hb** **by watching and waiting instead of transfusing RBCs** something you would usually do?

**EMOTION**

Would you feel worried about **managing a patient with borderline Hb** **by watching and waiting instead of transfusing RBCs**? If so, in what ways?

How would it influence your work stress to **manage a patient with borderline Hb** **by watching and waiting instead of transfusing RBCs**?
