# Supplementary material for: Using theories of behaviour to understand transfusion prescribing in three clinical contexts in two countries: Development work for an implementation trial
Source: Implement Sci. 2009 Oct 24;4:70. doi: 10.1186/1748-5908-4-70 (PMC2777847; doi:10.1186/1748-5908-4-70)
Supplement: Additional file 3 — Postal questionnaire used in UK study of intensive/critical care consultants. [file 1748-5908-4-70-S3.pdf]

## Part 4 - Background

**1** **Male** ☐

**Female** ☐

**2** **What is your job title and speciality?** ______

**3** **How long have you been a consultant?** ______ **yrs** ______ **months**

**4** **How many consultants work in your ICU?** ______

**5** **How many beds are there in your ICU?** ______

**6** **What percentage of your time is spent as an ICU consultant?** ______

**Is there any other comment you would like to make?**

______

**Date of completion** ______

**Please now return your completed questionnaire in the reply paid envelope provided**

If you wish to find out more about this study please contact:

Charlie Stockton
Research Assistant
Health Sciences Research Unit
University of Aberdeen
Health Sciences Building, 2nd Floor
Foresterhill
Aberdeen
AB25 2ZD

Tel: 01224 559035

Email: c.stockton@abdn.ac.uk



**Confidential**

Newcastle University

UNIVERSITY OF ABERDEEN

# *Doctors' use of blood and blood products*

The purpose of this questionnaire is to provide a better understanding of transfusion practice in Intensive Care Units.

We would like you to think about how you might…

***manage a patient with borderline haemoglobin by watching and waiting instead of transfusing red cells.***

When we say borderline haemoglobin, we are thinking about those patients where the decision about transfusing might need more thought. There will be patients you would definitely transfuse; patients you would definitely not transfuse; and patients where there is a grey area, where the decision about transfusing is less clear cut. **That is what we mean by borderline haemoglobin.**

All of the questionnaire items will refer to this hypothetical patient with borderline haemoglobin. We will ask you later what you consider to be borderline haemoglobin.

Most questions are answered by circling one number; a few require more time to answer.
Some questions may seem to be very similar but they are different and it is important to answer them all.
Please try not to take too long over each response as we would like to know your immediate views and experiences.
Your answers are completely confidential.

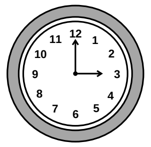


At first glance this questionnaire might seem long, however, our pilot testing with consultants suggests that, once you start it, you would be able to work through it quite quickly. We think it will take 15-20 minutes to complete.

I

## Part 1 - Questionnaire

**1 In general *managing a patient with borderline haemoglobin by watching and waiting instead of transfusing red cells* would:**

| | *Strongly disagree* | | | | | | *Strongly agree* |
|---|---|---|---|---|---|---|---|
| a) Decrease the patient's length of stay in the ICU | 1 | 2 | 3 | 4 | 5 | 6 | 7 |
| b) Improve the patient's clinical condition | 1 | 2 | 3 | 4 | 5 | 6 | 7 |
| c) Reduce costs and save resources | 1 | 2 | 3 | 4 | 5 | 6 | 7 |
| d) Reduce the risk of the patient contracting a transfusion related infection | 1 | 2 | 3 | 4 | 5 | 6 | 7 |

**2 In general how important do you consider these outcomes to be?**

| | *Unimportant* | | | | | | *Important* |
|---|---|---|---|---|---|---|---|
| a) Decreasing the patient's length of stay in the ICU | 1 | 2 | 3 | 4 | 5 | 6 | 7 |
| b) Improving the patient's clinical condition | 1 | 2 | 3 | 4 | 5 | 6 | 7 |
| c) Reducing costs and saving resources | 1 | 2 | 3 | 4 | 5 | 6 | 7 |
| d) Reducing the risk of the patient contracting a transfusionrelated infection | 1 | 2 | 3 | 4 | 5 | 6 | 7 |

| | *Strongly disagree* | | | | | | *Strongly agree* |
|---|---|---|---|---|---|---|---|
| **3 If I routinely *manage patients with borderline haemoglobin by watching and waiting instead of transfusing red cells* then:** | | | | | | | |
| a) On balance, my life as a critical care consultant will be easier in the long run | 1 | 2 | 3 | 4 | 5 | 6 | 7 |
| b) On balance, the consequences for me as a critical care consultant (e.g. stress, time etc.) will be worse in the long run | 1 | 2 | 3 | 4 | 5 | 6 | 7 |
| **4 If I m*anage patients with borderline haemoglobin by watching and waiting instead of transfusing red cells,* it is highly likely that they will be worse off** | 1 | 2 | 3 | 4 | 5 | 6 | 7 |
| **5 I feel under pressure to *manage patients with borderline haemoglobin by watching and waiting instead of transfusing red cells,* due to pressure from:** | | | | | | | |
| a) Colleagues within critical care | 1 | 2 | 3 | 4 | 5 | 6 | 7 |
| b) Guidelines and protocols | 1 | 2 | 3 | 4 | 5 | 6 | 7 |
| c) Published literature | 1 | 2 | 3 | 4 | 5 | 6 | 7 |
| d) Consultant colleagues from other specialities | 1 | 2 | 3 | 4 | 5 | 6 | 7 |
| e) Colleagues to whom I am handing over at the end of a shift | 1 | 2 | 3 | 4 | 5 | 6 | 7 |



**Scenario Three**

A 36 year old patient with no past medical history of note was admitted today with septic shock secondary to pneumonia. He presented yesterday and rapidly went into severe hypoxic respiratory failure and septic shock and was intubated, commenced on inotropic support and admitted to ICU. The main problem currently is septic shock and he is extremely ill with 4 sepsis induced organ system failures.

He has a PaO2 of 10kPa on an FiO2 of 0.9. He has been on pressure controlled ventilation with a PEEP of 15 cm H2O since admission. He is cardiovascularly unstable and is receiving high dose noradrenaline and adrenaline. He has some bleeding from his NG tube and is due an endoscopy today from the gastroenterologists.

Here are **today's** haematology results

| | Day 1 in ICU | **Today** (day 2) |
|---|---|---|
| Hb | 12.4 | 8.8 |
| WCC | 18.2 | 20.1 |
| Plat | 160 | 90 |

**3.1.1 If this was your patient would you transfuse this patient today? (Please circle)**

Yes Go to 2.4.2

No Go to 3.2

**3.1.2 How many units of red cells would you prescribe? (Please circle closest haemoglobin range).**

1 unit 2 units 3 units Other (please specify) ________________________________

**3.1.3 Which haemoglobin range would you aim for? (Please circle closest haemoglobin range).**

9.1 - 10.0g/dl 10.1 - 11.0g/dl 11.1 - 12.0g/dl Above 12.0g/dl

**If you decided to transfuse this patient, please go to the final part of the questionnaire**

**3.2 It is now later on the same day and the patient has had an upper GI endoscopy and injection of a bleeding stomach ulcer. He is still very unstable. You chose not to transfuse this patient earlier.**

Here are the current haematology results

| | Day 1 in ICU | Earlier (day 2) | Current (day 2) |
|---|---|---|---|
| Hb | 12.4 | 8.8 | 7.5 |
| WCC | 18.2 | 20.1 | 22.5 |
| Plat | 160 | 90 | 55 |

**3.2.1 If this was your patient would you transfuse this patient today? (Please circle)**

Yes Go to 3.22

No Which transfusion trigger would you use? _______g/dl

**3.2.2 How many units of red cells would you prescribe? (Please circle closest haemoglobin range).**

1 unit 2 units 3 units Other (please specify) ________________________________

**3.2.3 Which haemoglobin range would you aim for? (Please circle closest haemoglobin range).**

8.1 - 9.0g/dl 9.1 – 10g/dl 10.1 - 11.0g/dl 11.1 - 12.0g/dl Above 12.0g/dl

**2.2.3 Which haemoglobin range would you aim for? (Please circle closest haemoglobin range).**

9.1 – 10g/dl    10.1 - 11.0g/dl    11.1 - 12.0g/dl    Above 12.0g/dl

**If you decided to transfuse this patient, please go to Scenario 3**

**2.3 It is now day 4 and the patient's condition is similar. There is still no evidence of bleeding. You chose not to transfuse this patient yesterday.**

Here are **today's** haematology results

| | Day 1 in ICU | Day 2 in ICU | Day 3 in ICU | **Today** (day 4) |
|---|---|---|---|---|
| Hb | 10.9 | 9.8 | 8.6 | 7.7 |
| WCC | 10.2 | 12 | 11 | 10.7 |
| Plat | 160 | 170 | 163 | 191 |

**2.3.1 If this was your patient would you transfuse this patient today? (Please circle)**

Yes    Go to 2.3.2

No    Go to 2.4

**2.3.2 How many units of red cells would you prescribe? (Please circle closest haemoglobin range).**

1 unit    2 units    3 units    Other (please specify) ________________________________

**2.3.3 Which haemoglobin range would you aim for? (Please circle closest haemoglobin range).**

8.1 - 9.0g/dl    9.1 – 10.0g/dl    10.1 - 11.0g/dl    11.1 - 12.0g/dl    Above 12.0g/dl

**If you decided to transfuse this patient, please go to Scenario 3**

**2.4 It is now day 5 and the patient's condition is similar. There is still no evidence of bleeding. You chose not to transfuse this patient yesterday.**

Here are **today's** haematology results

| | Day 1 in ICU | Day 2 in ICU | Day 3 in ICU | Day in ICU | **Today** (day 5) |
|---|---|---|---|---|---|
| Hb | 10.9 | 9.8 | 8.6 | 7.7 | 6.9 |
| WCC | 10.2 | 12 | 11 | 10.7 | 9.9 |
| Plat | 160 | 170 | 163 | 191 | 192 |

**2.4.1 If this was your patient would you transfuse this patient today? (Please circle)**

Yes    Go to 2.4.2

No    Which transfusion trigger would you use? _______g/dl

**2.4.2 How many units of red cells would you prescribe? (Please circle closest haemoglobin range).**

1 unit    2 units    3 units    Other (please specify) ________________________________

**2.4.3 Which haemoglobin range would you aim for? (Please circle closest haemoglobin range).**

7.1 – 8.0g/dl    8.1 - 9.0g/dl    9.1-10.0g/dl    10.1 - 11.0g/dl    11.1 - 12.0g/dl    Above 12.0g/dl



| | | ***Strongly disagree*** | | | | | | ***Strongly agree*** |
|---|---|---|---|---|---|---|---|---|
| **6** | **When I first discover a patient has borderline haemoglobin, I have in mind to *manage them by watching and waiting instead of transfusing red cells*** | **1** | **2** | **3** | **4** | **5** | **6** | **7** |
| **7** | **I intend to *manage patients with borderline haemoglobin by watching and waiting instead of transfusing red cells*** | **1** | **2** | **3** | **4** | **5** | **6** | **7** |
| **8** | **Whether I *manage patients with borderline haemoglobin by watching and waiting instead of transfusing red cells* is entirely up to me** | **1** | **2** | **3** | **4** | **5** | **6** | **7** |
| **9** | **I am confident that I can *manage patients with borderline haemoglobin by watching and waiting instead of transfusing red cells* whenever I want to** | **1** | **2** | **3** | **4** | **5** | **6** | **7** |
| **10** | **I can overcome all obstacles, whatever they may be, in *managing a patient with borderline haemoglobin by watching and waiting instead of transfusing red cells*** | **1** | **2** | **3** | **4** | **5** | **6** | **7** |

**11 When you first discover a patient has borderline haemoglobin, out of 10 patients in this category how many would you intend to *manage by watching and waiting instead of transfusing red cells?***

**0    1    2    3    4    5    6    7    8    9    10**

**12 In general how confident are you in your ability to *manage a patient with borderline haemoglobin by watching and waiting instead of transfusing red cells?***

| **Not at all confident** | | | | | | **Extremely confident** |
|---|---|---|---|---|---|---|
| **1** | **2** | **3** | **4** | **5** | **6** | **7** |

**13 When I see patients with borderline haemoglobin, I automatically consider *managing them by watching and waiting instead of transfusing red cells* if their haemoglobin is:**

| | ***Strongly disagree*** | | | | | | ***Strongly agree*** |
|---|---|---|---|---|---|---|---|
| a) Rapidly declining (e.g. fallen 3g over 15 hours) | **1** | **2** | **3** | **4** | **5** | **6** | **7** |
| b) Slowly drifting down (e.g. fallen 3g over 4 days) | **1** | **2** | **3** | **4** | **5** | **6** | **7** |
| c) Stable | **1** | **2** | **3** | **4** | **5** | **6** | **7** |

| | Strongly disagree | | | | | | Strongly agree |
|---|---|---|---|---|---|---|---|
| **14 Before deciding to *manage a patient with borderline haemoglobin by watching and waiting instead of transfusing red cells* I take into account the opinions of:** | | | | | | | |
| a) Colleagues within critical care | 1 | 2 | 3 | 4 | 5 | 6 | 7 |
| b) Guidelines and protocols | 1 | 2 | 3 | 4 | 5 | 6 | 7 |
| c) Published literature | 1 | 2 | 3 | 4 | 5 | 6 | 7 |
| d) Consultant colleagues from other specialities | 1 | 2 | 3 | 4 | 5 | 6 | 7 |
| e) Colleagues to whom I am handing over at the end of a shift | 1 | 2 | 3 | 4 | 5 | 6 | 7 |
| **15 In general:** | | | | | | | |
| a) The benefits of *managing patients with borderline haemoglobin by watching and waiting instead of transfusing red cells* outweigh the harms | 1 | 2 | 3 | 4 | 5 | 6 | 7 |
| b) *Managing patients with borderline haemoglobin by watching and waiting instead of transfusing red cells* is more often the right thing to do than the wrong thing to do | 1 | 2 | 3 | 4 | 5 | 6 | 7 |
| c) When I *manage patients with borderline haemoglobin by watching and waiting instead of transfusing red cells* I feel pleased with my decision | 1 | 2 | 3 | 4 | 5 | 6 | 7 |
| **16 I have a clear plan of:** | | | | | | | |
| a) **How** I will *manage patients with borderline haemoglobin by watching and waiting instead of transfusing red cells* | 1 | 2 | 3 | 4 | 5 | 6 | 7 |
| b) **When** I will *manage patients with borderline haemoglobin by watching and waiting instead of transfusing red cells* | 1 | 2 | 3 | 4 | 5 | 6 | 7 |
| c) **Under what circumstances** I will *manage patients with borderline haemoglobin by watching and waiting instead of transfusing red cells* | 1 | 2 | 3 | 4 | 5 | 6 | 7 |

**17 If you have a plan, could you please describe it:**

**18 I am confident that I can *manage a patient with borderline haemoglobin by watching and waiting instead of transfusing red cells:***

| | Strongly disagree | | | | | | Strongly agree |
|---|---|---|---|---|---|---|---|
| a) Even if other clinicians are involved | 1 | 2 | 3 | 4 | 5 | 6 | 7 |
| b) When the patient's clinical condition deteriorates | 1 | 2 | 3 | 4 | 5 | 6 | 7 |
| c) With other senior clinicians standing next to me | 1 | 2 | 3 | 4 | 5 | 6 | 7 |



**Scenario Two**

A 75 year old patient with a history of severe coronary artery disease (previous myocardial infarction and angina on minimal exertion) was admitted to ICU yesterday. He presented to hospital two days ago with acute myocardial infarction and cardiogenic shock and rapidly went into severe hypoxic respiratory failure due to pulmonary oedema and was intubated and admitted to ICU. The main problem currently is cardiogenic shock.

He has a PaO2 of 9kPa on an FiO2 of 0.8. He has been on pressure controlled ventilation with a PEEP of 10 cmH2O since admission. He is cardiovascularly unstable and is receiving inotropic support with dobutamine at 20µg/kg/min. He shows no signs of clinical bleeding. His mean arterial pressure is 65 mmHg and there is evidence of ongoing myocardial ischaemia on the ECG. His coronary lesions are not seen as being amenable to coronary intervention.

Here are **today's** haematology results

| | Day 1 in ICU | **Today** (day 2) |
|---|---|---|
| Hb | 10.9 | 9.8 |
| WCC | 10.2 | 12 |
| Plat | 160 | 170 |

**2.1.1 If this was your patient would you transfuse this patient today? (Please circle)**

Yes Go to 2.1.2

No Go to 2.2

**2.1.2 How many units of red cells would you prescribe? (Please circle closest haemoglobin range).**

1 unit 2 units 3 units Other (please specify) ________________________________

**2.1.3 Which haemoglobin range would you aim for? (Please circle closest haemoglobin range).**

10.1 – 11.0g/dl 11.1 - 12.0g/dl Above 12.0g/dl

**If you decided to transfuse this patient, please go to Scenario 3**

**2.2 It is now day 3 and the patient's condition is still very unstable. They still require full ventilation and high FiO2 and are still on inotropic support. You chose not to transfuse this patient yesterday.**

Here are **today's** haematology results

| | Day 1 in ICU | Day 2 in ICU | **Today** (day 3) |
|---|---|---|---|
| Hb | 10.9 | 9.8 | 8.6 |
| WCC | 10.2 | 12 | 11 |
| Plat | 160 | 170 | 163 |

**2.2.1 If this was your patient would you transfuse this patient today? (Please circle)**

Yes Go to 2.2.2

No Go to 2.3

**2.2.2 How many units of red cells would you prescribe? (Please circle closest haemoglobin range).**

1 unit 2 units 3 units Other (please specify) ________________________________

**1.2.1** **If this was your patient would you transfuse this patient today? (Please circle)**

Yes Go to 1.2.2

No Go to 1.3

**1.2.2** **How many units of red cells would you prescribe? (Please circle closest haemoglobin range).**

1 unit 2 units 3 units Other (please specify) ________________________________

**1.2.3** **Which haemoglobin range would you aim for? (Please circle closest haemoglobin range).**

8.1 – 9.0g/dl 9.1 - 10.0g/dl 10.1 - 11.0g/dl 11.1 - 12.0g/dl Above 12.0g/dl

***If you decided to transfuse this patient, please go to Scenario 2***

**1.3** **It is now day 8 and the patient's condition is similar and they are still weaning from the ventilator with stable renal function (140 micromol/l). You chose not to transfuse this patient two days ago.**

Here are **today's** haematology results

| | Pre-op | Day 1 in ICU | Day 2 in ICU | Day 3 in ICU | Day 4 in ICU | Day 5 in ICU | Day 6 in ICU | Day 7 in ICU | **Today** (day 8) |
|---|---|---|---|---|---|---|---|---|---|
| Hb | 12.9 | 9.1 | 9.1 | 9 | 8.9 | 8.3 | 7.9 | 7.4 | 6.9 |
| WCC | 8.8 | 18.6 | 18.5 | 16 | 15.7 | 12 | 11.8 | 10.1 | 9.9 |
| Plat | 358 | 250 | 190 | 130 | 103 | 110 | 120 | 140 | 180 |

**1.3.1** **If this was your patient would you transfuse this patient today? (Please circle)**

Yes Go to 1.3.2

No If no which transfusion trigger would you use-________g/dl

**1.3.2** **How many units of red cells would you prescribe? (Please circle closest haemoglobin range).**

1 unit 2 units 3 units Other (please specify) ________________________________

**1.3.3** **If you were transfusing this patient, which haemoglobin range would you aim for? (Please circle closest haemoglobin range).**

7.1 – 8.0g/dl 8.1 - 9.0g/dl 9.1 - 10.0g/dl 10.1 - 11.0g/dl 11.1 - 12.0g/dl Above 12.0g/dl



| | *Strongly disagree* | | | | | | *Strongly agree* |
|---|---|---|---|---|---|---|---|
| **19** **I would be more likely to *manage a patient with borderline haemoglobin by watching and waiting instead of transfusing red cells* if the patient:** | | | | | | | |
| a) Has ischaemic heart disease | **1** | **2** | **3** | **4** | **5** | **6** | **7** |
| b) Is due to be discharged from the ICU | **1** | **2** | **3** | **4** | **5** | **6** | **7** |
| c) Is over 55 years of age | **1** | **2** | **3** | **4** | **5** | **6** | **7** |
| d) Has stable haemoglobin levels | **1** | **2** | **3** | **4** | **5** | **6** | **7** |
| **20** **I would be more likely to *manage a patient with borderline haemoglobin by transfusing red cells* if the patient:** | | | | | | | |
| a) Has ischaemic heart disease | **1** | **2** | **3** | **4** | **5** | **6** | **7** |
| b) Is due to be discharged from the ICU | **1** | **2** | **3** | **4** | **5** | **6** | **7** |
| c) Is over 55 years of age | **1** | **2** | **3** | **4** | **5** | **6** | **7** |
| d) Has stable haemoglobin levels | **1** | **2** | **3** | **4** | **5** | **6** | **7** |

## Part 2 - About Transfusion

**1 There are different opinions about what constitutes borderline haemoglobin. In your opinion, what constitutes borderline haemoglobin?**

**2 What are the 3 most frequent adverse risks related to transfusion from the following list?**

*Please tick the 3 most frequent:*

a) ABO incompatible transfusions ☐

b) Errors in the process/administration of blood ☐

c) Acute lung injury ☐

d) Acute transfusion reactions (e.g. anaphylaxis) ☐

e) Transfusion transmitted viral infection ☐

f) CJD ☐

g) Transfusion associated graft versus host disease ☐

**3 Out of the above list of adverse events, which is the most likely to cause mortality in ICU?**

*Please circle the one that applies:*

**a b c d e f g**

**4 What is the average cost of a unit of red cells in the UK?**

*Please tick the one that applies:*

a) £30 ☐

b) £130 ☐

c) £230 ☐

**5 The Canadian TRICC trial (Hébert et al., 1999, A multicenter, randomized, controlled clinical trial of transfusion requirements in critical care. *New England Journal of Medicine. 409-417*) compared which of the following haemoglobin thresholds for transfusion triggers?**

*Please tick the one that applies:*

a) 6g/dl versus 9g/dl ☐

b) 7g/dl versus 10g/dl ☐

c) 8g/dl versus 10g/dl ☐

d) Don't know ☐

e) Other (please specify) ☐



## Part 3 - How would you manage this patient?

**Scenario One**

A previously fit and well 65 year old male has been ventilated in your ICU for a number of days. He developed post-operative pneumonia following an emergency laparotomy for perforated diverticulum. The main problem currently is failure to wean from the ventilator.

He has a $PaO_2$ of 12kPa on an $FiO_2$ of 0.4. He has been on pressure support ventilation of 15 cm $H_2O$ with a PEEP of 5 cm $H_2O$ for the past 2 days. He becomes tachypnoeic and distressed when attempts are made to decrease inspiratory support.

Cardiovascularly stable, the patient is not receiving inotropic or vasopressor therapy and shows no signs of clinical bleeding. His mean arterial pressure is 75 mmHg and there is no evidence of myocardial ischaemia on the ECG.

He developed acute renal failure (peak creatinine 350 micromol/l) on day 3, but this is now resolving and he did not require renal replacement therapy (current creatinine 180 micromol/l; urine output 50-80mls per hour). Bilirubin is 26 micromol/l. He has no coagulopathy.

Here are **today's** haematology results

| | Pre-op | Day 1 in ICU | Day 2 in ICU | Day 3 in ICU | **Today** (day 4) |
|---|---|---|---|---|---|
| Hb | 12.9 | 9.1 | 9.1 | 9 | 8.9 |
| WCC | 8.8 | 18.6 | 18.5 | 16 | 15.7 |
| Plat | 358 | 250 | 190 | 130 | 103 |

**1.1.1 If this was your patient would you transfuse this patient today? (Please circle)**

Yes Go to 1.1.2

No Go to 1.2

**1.1.2 How many units of red cells would you prescribe? (Please circle closest haemoglobin range).**

1 unit 2 units 3 units Other (please specify) ________________________________

**1.1.3 Which haemoglobin range would you aim for? (Please circle closest haemoglobin range).**

9.1 – 10g/dl 10.1 - 11.0g/dl 11.1 - 12.0g/dl Above 12.0g/dl

***If you decided to transfuse this patient, please go to Scenario 2***

**1.2 It is now day 6 and the patient's condition is similar and they are still weaning from the ventilator with stable renal function (160 micromol/l). You chose not to transfuse this patient two days ago.**

Here are **today's** haematology results

| | Pre-op | Day 1 in ICU | Day 2 in ICU | Day 3 in ICU | Day 4 in ICU | Day 5 in ICU | **Today** (day 6) |
|---|---|---|---|---|---|---|---|
| Hb | 12.9 | 9.1 | 9.1 | 9 | 8.9 | 8.3 | 7.9 |
| WCC | 8.8 | 18.6 | 18.5 | 16 | 15.7 | 12 | 11.8 |
| Plat | 358 | 250 | 190 | 130 | 103 | 110 | 120 |
